# Development of a novel method for the quantification of tyrosine 39 phosphorylated α- and β-synuclein in human cerebrospinal fluid

**DOI:** 10.1186/s12014-020-09277-8

**Published:** 2020-05-04

**Authors:** Chan Hyun Na, Gajanan Sathe, Liana S. Rosenthal, Abhay R. Moghekar, Valina L. Dawson, Ted M. Dawson, Akhilesh Pandey

**Affiliations:** 1 Neurodegeneration Program, Institute for Cell Engineering, Johns Hopkins University School of Medicine, Baltimore, MD 21205 USA; 2 Department of Neurology, Johns Hopkins University School of Medicine, Baltimore, MD 21205 USA; 3 Diana Helis Henry Medical Research Foundation, New Orleans, LA 70130 USA; 4 Department of Biological Chemistry, McKusick-Nathans Institute of Genetic Medicine, Johns Hopkins University School of Medicine, Baltimore, MD 21205 USA; 5 Department of Physiology, Johns Hopkins University School of Medicine, Baltimore, MD 21205 USA; 6 Solomon H. Snyder Department of Neuroscience, Johns Hopkins University School of Medicine, Baltimore, MD 21205 USA; 7 Department of Pharmacology and Molecular Sciences, Johns Hopkins University School of Medicine, Baltimore, MD 21205 USA; 8 Manipal Academy of Higher Education (MAHE), Manipal, 576104 Karnataka India; 9 Present Address: Center for Molecular Medicine, National Institute of Mental Health and Neurosciences (NIMHANS), Hosur Road, Bangalore, 560 029 India; 10 Present Address: Laboratory Medicine and Pathology, Mayo Clinic, Rochester, MN 55902 USA

**Keywords:** Parkinson’s disease, α-Synuclein, β-Synuclein, Phosphotyrosine, Cerebrospinal fluid, Parallel reaction monitoring

## Abstract

**Background:**

Parkinson’s disease (PD) is the second most prevalent neurodegenerative disorder. Biomarkers that can help monitor the progression of PD or response to disease-modifying agents will be invaluable in making appropriate therapeutic decisions. Further, biomarkers that could be used to distinguish PD from other related disorders with PD-like symptoms will be useful for accurate diagnosis and treatment. C-Abl tyrosine kinase is activated in PD resulting in increased phosphorylation of the tyrosine residue at position 39 (Y39) of α-synuclein (α-syn) (pY39 α-syn), which contributes to the death of dopaminergic neurons. Because pY39 α-syn may be pathogenic, monitoring pY39 α-syn could allow us to diagnose presymptomatic PD and help monitor disease progression and response to treatment. We sought to investigate if increased phosphorylation of pY39 α-syn can be detected in the cerebrospinal fluid (CSF) of PD patients by targeted mass spectrometry.

**Methods:**

Here, we report a two-step enrichment method in which phosphotyrosine peptides were first enriched with an anti-phosphotyrosine antibody followed by a second round of enrichment by titanium dioxide (TiO_2_) beads to detect EGVLpYVGSK sequence derived from tyrosine 39 region of α- and β-synuclein (αβ-syn). Accurate quantification was achieved by adding a synthetic heavy version of pY39 αβ-syn peptide before enzymatic digestion.

**Results:**

Using the developed enrichment methods and optimized parallel reaction monitoring (PRM) assays, we detected pY39 αβ-syn peptide in human CSF and demonstrated that the ratio of pY39 αβ-syn to Y39 αβ-syn was significantly increased in the CSF of patients with PD.

**Conclusions:**

We anticipate that this optimized two-step enrichment-based PRM detection method will help monitor c-Abl activation in PD patients and can also be used to quantify other phosphotyrosine peptides of low abundance in biological samples.

## Introduction

Although the exact pathogenic mechanism of PD has yet to be established, α-syn is an important mediator, and moreover, the phosphorylation of α-syn may contribute to the pathogenesis via increased aggregation and toxicity [1, 2]. Recently, pY39 α-syn has been shown to be closely correlated to disease severity and progression [1, 3, 4]. Thus, pY39 α-syn levels in the brain could potentially serve as a marker for presymptomatic diagnosis, disease progression, and therapeutic response. Since pY39 α-syn is increased in the brain of PD patients, we postulated that this increased phosphorylation could be reflected in the CSF [5].

For targeted detection and quantitation of known proteins or post-translational modifications (PTMs) on those proteins, PRM mass spectrometry (PRM-MS) has been widely used [6, 7]. To quantify pY39 α-syn using PRM-MS, pY39 αβ-syn peptide, EGVLpYVGSK will be monitored. Although pY39 αβ-syn peptide is shared between α-syn and β-syn, we reasoned that the peptide can still serve as a PD biomarker unless the phosphorylation level of β-syn changes to the opposite direction to that of α-syn in PD. When the abundance of a target protein is too low, the target protein or the derived peptide sometimes can be detected using various enrichment methods such as immunoprecipitation or affinity purification of target proteins or peptides [8–10]. Here, we report a method specifically developed to detect pY39 αβ-syn peptide in human CSF combining an enrichment method with the PRM-MS approach. Because the abundance of pY39 αβ-syn in CSF is too low to be detected even with a conventional enrichment method, we developed a two-step enrichment approach using an anti-phosphotyrosine antibody and TiO_2_ beads followed by PRM-MS analysis for detection and quantitation of pY39 αβ-syn peptide. This method allowed us to establish that the ratio of pY39 αβ-syn to Y39 αβ-syn peptides in the CSF can serve as a potential biomarker for the diagnosis and prognosis of PD. Furthermore, this method is applicable to the extremely sensitive detection of other phosphotyrosine peptides as well.

## Methods

### Collection of CSF samples

The CSF specimens were collected from normal pressure hydrocephalus (NPH) patients, PD patients or cognitively normal healthy control individuals evaluated by investigators at the Johns Hopkins Hospital. The CSF samples from NPH patients were used for method optimization. The CSF samples from PD or control individuals were used to compare pY39 αβ-syn peptide levels between the two groups. The individuals who are cognitively normal or show PD symptoms were diagnosed after extensive clinical and cognitive testing. All PD patients met the UK Brain Bank criteria for PD diagnosis [11]. After the collection of CSF samples by lumbar puncture, the samples were centrifuged for 10 min at 1500×*g*, aliquoted, and stored at − 80 °C within 1 h of acquisition. The demographic and clinical characteristics of the PD patients and control individuals are shown in Additional file 1: Table S1.

### Enrichment of pY39 αβ-syn peptide only with anti-phosphotyrosine antibody

Approximately ~ 5.5 mg of proteins derived from 9 ml of CSF were lysed in 4 M urea and 50 mM triethylammonium bicarbonate (TEAB) followed by a reduction with 10 mM dithiothreitol for 1 h at room temperature (RT) and alkylation with 30 mM iodoacetamide for 30 min at RT in the dark. The proteins were then digested with an endoproteinase Lys-C (1:100; Wako Chemicals, Richmond, VA) by incubating at RT for 3 h. Subsequently, trypsin digestion was conducted by diluting the urea concentration to 2 M by adding 1 volume of 50 mM TEAB followed by adding sequencing-grade trypsin (1:50; Promega, Madison, WI) and incubating at 37 °C overnight. The peptide samples were desalted with C_18_ Sep-Pak (Waters Corporation, Milford, MA) and freeze-dried. The pY39 αβ-syn endogenous peptide (EGVLpYVGSK) was enriched by performing phosphotyrosine peptide enrichment with PTMScan pY1000 antibody according to the manufacturer’s instruction (Cell Signaling Technology, Danvers, MA). Briefly, the ~ 2.75 mg of CSF peptides derived from 9 ml of CSF was reconstituted in 1.4 ml of immunoaffinity purification buffer (IAP, 50 mM MOPS, pH 7.2, 10 mM Na_2_HPO_4_ and 50 mM NaCl). The peptide solution was cleared by centrifugation for 5 min at 10,000×*g* at 4 °C, and the supernatant was subject to the phosphotyrosine enrichment. After washing 40 µl of phosphotyrosine agarose beads three times with PBS, the CSF peptide solution was added to the washed beads followed by incubation at 4^o^ C for 2 h with rotation. Subsequently, the supernatant was removed, and the beads were washed thrice with 1 ml of IAP buffer and twice with 1 ml of ice-cold water. The bound phosphotyrosine peptides were eluted by adding 55 µl of 0.15% trifluoroacetic acid (TFA) and incubated at RT for 10 min. After incubation, the tube was centrifuged at 2000×*g* for 1 min and the solution was transferred to a new tube. This elution was repeated once again with 50 µl 0.15% TFA. Twenty fmol of synthetic heavy (^13^C_6_, ^15^N_2_-lysine) pY39 αβ-syn peptide was added followed by desalting with C_18_ StageTip. The eluted peptides were then dried using a SpeedVac followed by reconstitution in 15 µl of 0.1% formic acid prior to mass spectrometry analysis.

### Enrichment of pY39 αβ-syn peptide both with anti-phosphotyrosine antibody and TiO_2_ beads

For quantification of pY39 αβ-syn peptides from 1 ml (~ 0.6 mg of proteins) of CSF samples from PD patients or control samples with both PTMScan pY1000 antibody and TiO2, 20 fmol of synthetic heavy pY39 αβ-syn peptide was added to CSF. CSF proteins were lysed in 4 M urea and 50 mM TEAB followed by a reduction with 10 mM dithiothreitol for 1 h at RT and alkylation with 30 mM iodoacetamide for 30 min at RT in the dark. The proteins were then digested with an endoproteinase Lys-C (1:100; Wako Chemicals, Richmond, VA) by incubating at RT for 3 h. Sequentially trypsin digestion was conducted by diluting the urea concentration to 2 M by adding 1 volume of 50 mM TEAB followed by adding sequencing-grade trypsin (1:50; Promega, Madison, WI) and incubating at 37 °C overnight. The peptide samples were desalted with C_18_ Sep-Pak (Waters Corporation, Milford, MA) and freeze-dried. The synthetic heavy and endogenous pY39 αβ-syn peptides were enriched by performing phosphotyrosine peptide enrichment with PTMScan pY1000 antibody according to the manufacturer’s instruction with minor modifications (Cell Signaling Technology, Danvers, MA). Briefly, the ~ 0.3 mg of CSF peptides derived from 1 ml of CSF was reconstituted in 200 µl of IAP buffer. The peptide solution was cleared by centrifugation for 5 min at 10,000×*g* at 4 °C and the supernatant was subjected to the phosphotyrosine enrichment. After washing 20 µl of phosphotyrosine agarose beads three times with PBS, the CSF peptide solution was added to the washed beads followed by incubation at 4 °C for 2 h with rotation. Subsequently, the supernatant was removed and the beads were washed once with ice-cold water. The bound phosphotyrosine peptides were eluted by adding 55 µl of 0.15% TFA and incubated at RT for 10 min. After incubation, the tube was centrifuged at 2000×*g* for 1 min and the solution was transferred to a new tube. This elution was repeated once again with 50 µl 0.15% TFA. The eluate was dried using a SpeedVac and the phosphorylated peptides were enriched again using TiO_2_ beads as described previously [12]. Briefly, 0.6 mg of TiO_2_ beads (Titansphere) resuspended in 40 µl of binding buffer (65% acetonitrile (ACN) and 2% TFA) were added to the peptides followed by incubation at RT for 20 min with shaking at 1400 rpm. The peptides were transferred to a C_8_ StageTip and centrifuged at 2000×*g* for 2 min. Two hundred µl of the washing buffer (65% ACN and 0.1% TFA) was added and centrifuged at 2000×*g* for 5 min. This washing was repeated once again. The phosphopeptides were eluted by adding 40 µl of elution buffer (1% NH_4_OH and 40% ACN) and centrifuging at 200×*g* for 2 min. The eluted peptides were then dried using a SpeedVac followed by reconstitution in 15 µl of 0.1% formic acid prior to mass spectrometry analysis.

### Calculation of the limits of detection and quantification

The limits of detection (LOD) were calculated as: LOD = µ_B_ + t_(1-β)_ (σ_B_ + σ_S_)/√n, where µ_B_ is the estimated mean of blank samples, t_(1-β)_ is 95 percentile of the standard t distribution on f degrees of freedom, σ_B_ is the standard deviation of the blank samples, σ_S_ is the standard deviation of the low concentration samples, and n is the number of replicates. The limits of quantification (LOQ) were estimated as 3x LOD [13].

### Detection of Y39 αβ-syn peptide

To normalize the amount of pY39 αβ-syn peptide in each sample based on the Y39 αβ-syn peptide present in each sample, the amount of Y39 αβ-syn peptide in each sample was also measured. Twenty fmol of heavy (^13^C_6_, ^15^N_2_-lysine) Y39 αβ-syn peptide (EGVLYVGSK*) for the quantification of the endogenous Y39 αβ-syn peptide were added to 5 µg of CSF peptides followed by desalting with C_18_ StageTip and LC–MS/MS analysis.

### LC–MS/MS analysis

The prepared peptides were analyzed on an Orbitrap Fusion Lumos Tribrid Mass Spectrometer coupled to an EASY-nLC 1200 nano-flow liquid chromatography system (Thermo Fisher Scientific). The peptides from each fraction were reconstituted in 15 μl of 0.1% formic acid and loaded onto an Acclaim PepMap100 Nano-Trap Column (100 μm × 2 cm, Thermo Fisher Scientific) packed with 5 μm C_18_ particles at a flow rate of 5 μl per min. The flow rate employed was 250 nl/min using a linear gradient of 10% to 35% solvent B (0.1% formic acid in 95% acetonitrile) over 45 minutes on an EASY-Spray column (50 cm × 75 µm ID, Thermo Fisher Scientific) packed 2 µm C_18_ particles (Thermo Fisher Scientific), which was fitted with an EASY-Spray ion source operated at a voltage of 2.7 kV. Mass spectrometry analysis was completed in a data-dependent manner with a full scan in the mass-to-charge ratio (*m/z*) range of 350 to 1550 followed by targeted MS2. MS1 was measured at a resolution of 120,000 (at *m/z* of 200). MS2 scan was acquired by fragmenting precursor ions using the higher-energy collisional dissociation (HCD) method and detected at a mass resolution of 30,000 (at *m/z* of 200). Automatic gain control was set to 500,000 and 100,000 ions for MS1 and MS2, respectively. The maximum ion injection time for MS1 was set to 100 ms. Maximum ion times for MS2 were set to 2500 and 500 ms for pY39 and Y39 αβ-syn peptides, respectively. HCD normalized collisional energy (NCE) was set to 25, if not specified. The precursor isolation window was set to 1.6 *m/z*. Internal calibration was carried out using the lock mass option (*m/z* 445.1200025) from ambient air. For the light and heavy pY39 αβ-syn peptides, *m/z* 516.244 and *m/z* 520.251 were monitored, respectively. For the light and the heavy Y39 αβ-syn peptides, *m/z* 476.261 and *m/z* 480.268 were monitored, respectively.

### Data analysis

The quantification of relative peptide abundance was performed using Skyline software [14]. The levels of pY39 αβ-syn were normalized by Y39 αβ-syn peptide.

## Results

**Fig. 1:**
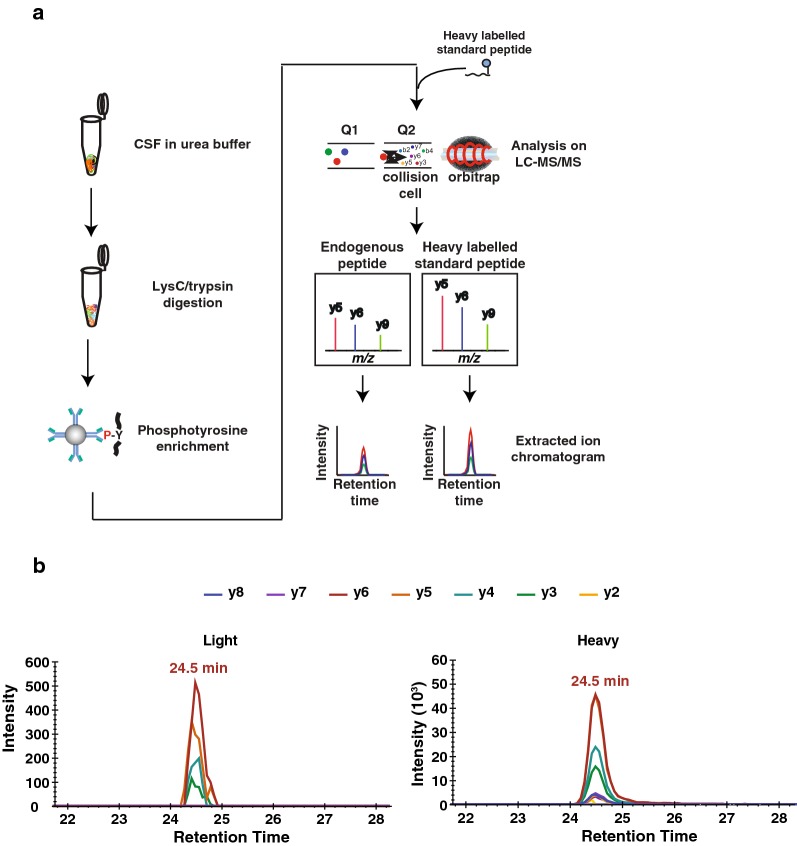
The schematic diagram for the research strategy and the detection of endogenous pY39 αβ-syn peptides. **a** The experimental strategy for pY39 αβ-syn peptide enrichment using an anti-phosphotyrosine antibody. CSF proteins were digested with Lys-C and trypsin followed by phosphotyrosine peptide enrichment. To validate the detection of the endogenous pY39 αβ-syn peptide, a heavy pY39 αβ-syn standard peptide was added before PRM analysis. The endogenous light and heavy standard pY39 αβ-syn peptides were monitored under PRM mode followed by a quantification using Skyline software. **b** The extracted chromatogram of *y* ion series from either endogenous or heavy standard pY39 αβ-syn peptide

To detect pY39 αβ-syn peptide in CSF samples, we initially tried to detect it directly from the digests of CSF samples using PRM but were not able to detect it. Subsequently, we tried an enrichment of α-syn protein using an anti-α-syn antibody or enrichment of phosphopeptides using TiO_2_ or Immobilized metal affinity chromatography (IMAC). However, none of these enrichment methods were successful in detecting pY39 αβ-syn peptide. We reasoned that because pY39 αβ-syn peptide is a tyrosine-phosphorylated peptide, we could further reduce peptide complexity by performing phosphotyrosine peptide enrichment to remove phosphoserine and phosphothreonine peptides, which constitute the majority of phosphopeptides. Using this procedure, we were able to detect the endogenous pY39 αβ-syn peptide from 9 ml of CSF (Fig. 1a, b). Because 9 ml of CSF is not practical volume in most cases for biomarker detection, we further optimized our methods to reduce the CSF volume required for the analysis.

### Optimizing the detection of pY39 αβ-syn peptide

**Fig. 2:**
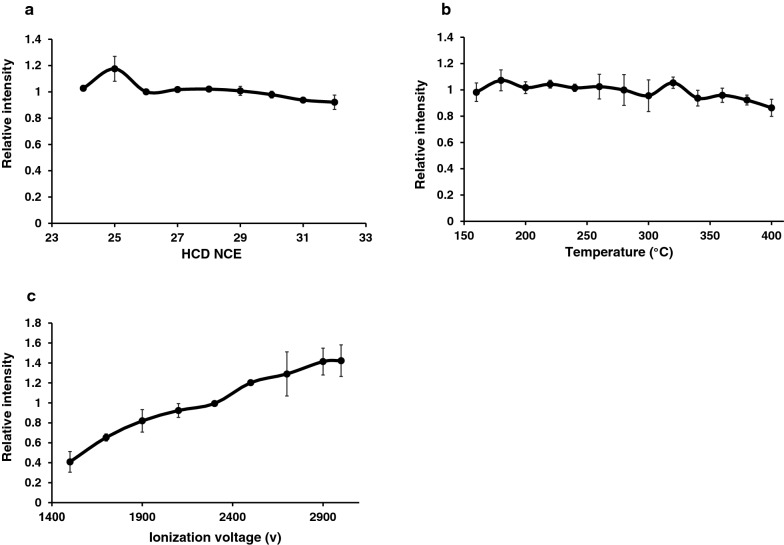
Optimization of mass spectrometry parameters for pY39 peptide detection. **a** The relative intensity of the standard pY39 αβ-syn peptide with different HCD NCEs. **b** The relative intensity of the standard pY39 αβ-syn peptide with different ion transfer tube capillary temperatures. **c** The relative intensity of the standard pY39 αβ-syn peptide with different ESI voltages

To improve our detection sensitivity, we systematically evaluated the effect of HCD NCE energy, ion transfer capillary temperature and ESI spray voltage on detection. For the HCD NCE evaluation, the HCD NCE value was increased from 24 to 32. The pY39 αβ-syn peptide showed the highest intensity at 25 of HCD NCE (Fig. 2a). Next, we evaluated the temperature of the ion transfer capillary increasing it from 160 to 400 °C and pY39 αβ-syn peptide showed the highest intensity at 180 °C (Fig. 2b). We then evaluated ESI voltage optimization by increasing from 1500 to 3000. pY39 αβ-syn peptide showed the highest intensity at 3000 V (Fig. 2c). Interestingly, the intensity at 3000 V was > 3.5-fold compared to the one observed at 1500 V. Although the HCD NCE and the ion transfer capillary temperature did not change the intensity of pY39 αβ-syn peptide significantly, the ESI voltage seemed to be critical in increasing the sensitivity. We applied the optimized parameters for the detection of pY39 αβ-syn peptide except that we opted to use 2700 V instead of 3000 V for preserving column stability. A calibration curve with these optimized parameters permitted us to detect the target peptide at sub-attomole levels. The LOD and LOQ were 0.44 and 1.32 attomoles, respectively. The coefficient of variations (CV) was calculated as 26.19% at 1 attomole, 4.45% at 10 attomoles, 4.84% at 100 attomoles, 10.03% at 1 femtomole, 4.45% at 10 femtomoles and 8.93% at 100 femtomoles. The average CV of the 6 concentrations was 9.81% (Additional file 2: Figure S1).

### Development of an enrichment method for pY39 αβ-syn peptides

**Fig. 3:**
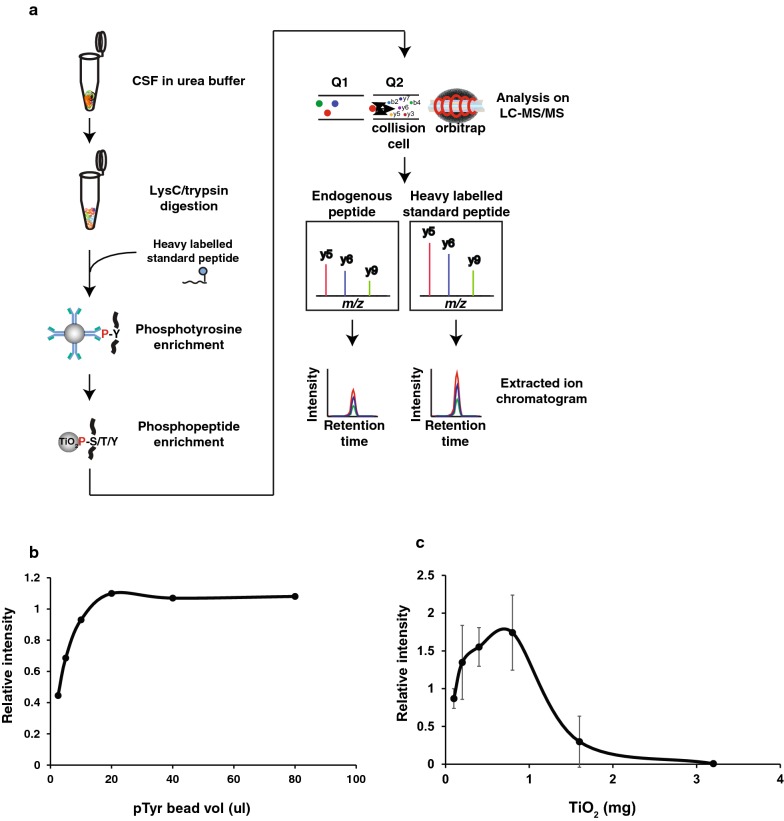
Two-step enrichment strategy and the optimization of the two-step enrichment experiment conditions. **a** The experimental strategy for pY39 αβ-syn peptide enrichment using the two-step enrichment approach in which phosphotyrosine peptides were enriched followed by total phosphopeptides were enriched using TiO_2_ beads. CSF proteins were digested with Lys-C and trypsin. To monitor the enrichment efficiency, heavy standard pY39 αβ-syn peptide was added before the two-step enrichment and the enrichments were conducted. The endogenous light and heavy standard pY39 αSyn were monitored under PRM mode followed by quantification using Skyline software. **b** Different volumes of agarose beads coupled with anti-phosphotyrosine antibodies were incubated with the target peptides to investigate the best ratio of anti-phosphotyrosine agarose beads to the target peptides. **c** Different amounts of TiO_2_ beads were incubated with the target peptides to investigate the best ratio of TiO_2_ beads to the target peptides

To detect the pY39 αβ-syn peptide from an even smaller volume of CSF, we optimized the enrichment method as well. Even after the enrichment of phosphotyrosine peptides, 80–90% of peptides were still non-phosphorylated ones interfering with the detection of target peptides. Thus, the second step of TiO_2_-based phosphopeptide enrichment should further remove the non-phosphorylated peptides, and thereby, we expected that it would increase the sensitivity of detecting target peptides (Fig. 3a). Thus, we developed a two-step enrichment method by combining the antibody-based phosphotyrosine peptide enrichment method with the TiO_2_-based phosphopeptide enrichment method. To optimize this two-step detection method, we first evaluated the ratio of phosphotyrosine antibody-conjugated agarose beads to the input peptide amount. One femtomole of heavy pY39 αβ-syn peptide was incubated with various volumes of antibody-conjugated beads ranging from 2.5 ul to 80 ul. The relative intensity of the heavy pY39 αβ-syn peptide showed saturation at 20 µl of the agarose beads (Fig. 3b). We next optimized the ratio of TiO_2_ beads to input peptide amount. For this, 2 femtomoles of heavy pY39 αβ-syn peptide was incubated with various amounts of TiO_2_ beads ranging from 0.1 mg to 3.2 mg. The pY39 αβ-syn peptide showed the highest intensity at 0.8 mg of TiO_2_ beads (Fig. 3c).

### Measurement of pY39 αβ-syn peptide in CSF samples from PD and control individuals

**Fig. 4:**
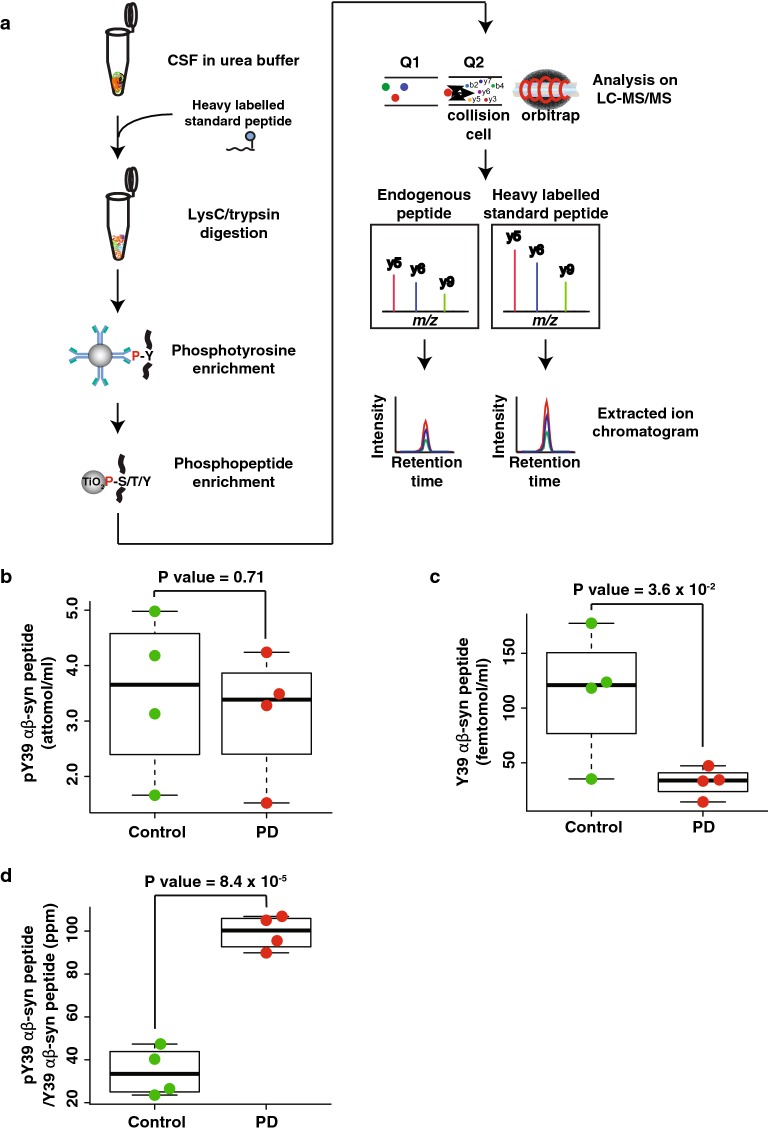
Quantification of pY39 αβ-syn peptide in CSF samples from PD and control individuals. **a** The experimental strategy for pY39 αβ-syn peptide enrichment from PD and control CSFs using the two-step enrichment approach. CSF proteins were digested with Lys-C and trypsin followed by the two-step enrichment. To minimize experimental biases, heavy standard pY39 αβ-syn peptide was added before starting the experiment. The endogenous light and heavy standard pY39 αβ-syn peptides were monitored under PRM mode followed by quantification using Skyline software. **b** The abundances of pY39 αβ-syn peptide in PD and control CSFs. **c** The abundances of Y39 αβ-syn peptide in PD and control CSFs. **d** The relative abundance of pY39 to Y39 αβ-syn peptide in PD and control CSFs

Since the goal of this study was to quantify the endogenous pY39 αβ-syn peptide in CSF samples from PD patients and control individuals, we applied the optimized mass spectrometry parameters and sample preparation procedure to detect the pY39 αβ-syn peptides from 4 PD and 4 control individuals as shown in Fig. 4a. To minimize any experimental bias and maximize the accuracy of quantification, synthetic heavy pY39 αβ-syn peptide was added to all CSF samples at the beginning of the sample preparation step [15]. When pY39 αβ-syn peptide levels in 1 ml of CSF samples from PD patients were compared to the ones from controls, there was no statistically significant difference (Fig. 4b and Additional files 3: Table S2, 4). Because it is already known that total α-syn levels are decreased in PD patients, we postulated that the levels of pY39 αβ-syn peptide normalized to the ones of Y39 αβ-syn peptide might help distinguish PD patients from controls. For this, we first measured the Y39 αβ-syn levels by spiking synthetic heavy Y39 αβ-syn peptide (Additional file 5: Figure S2). As we expected, Y39 αβ-syn peptide in CSF from PD patients showed a decreased abundance (Fig. 4c). Most importantly, the ratio of pY39 αβ-syn to Y39 αβ-syn peptides in CSF from PD patients showed a noticeable increase (2.5 fold) with statistical significance (P value = 8.4 × 10^−5^) (Fig. 4d). These results are potentially promising and will have to be validated in a larger cohort.

## Discussion

In this study, we developed a two-step enrichment method to detect endogenous pY39 αβ-syn peptide in a sensitive manner from CSF samples using PRM-MS. This approach enabled us to detect the phosphorylated target peptide present at attomole levels per ml of CSF. The enrichment efficiency of phosphotyrosine peptides using the anti-phosphotyrosine antibody-conjugated beads in the first enrichment was usually < 15% owing to non-specifically bound peptides even after 5 washes. These non-specifically bound peptides increase the noise in PRM-MS experiments thereby reducing the detection sensitivity. These non-specifically bound peptides can be removed by more extensive washing but it will result in the loss of the target peptide at the same time. For this reason, we chose to remove the non-specifically bound peptides by the second enrichment step using TiO_2_ beads instead of washing the beads stringently losing the target peptide. Using this strategy, we were able to improve the detection sensitivity of the target peptide to the level of attomole per ml. While the endogenous pY39 αβ-syn peptide levels alone did not show a statistically significant difference between PD and control CSFs, the relative abundance of pY39 over Y39 αβ-syn peptides was strikingly different between the two groups with statistical significance. Our results suggest that the stoichiometry of tyrosine phosphorylation on the residue 39 of αβ-syn might be increased in patients with PD, although how much phosphorylation was derived from α-syn still remains to be elucidated. The development of this method now makes it possible to test the utility of the pY39 over Y39 αβ-syn peptide ratio as a potential readout for c-Abl activity as well. In addition, this method is broadly applicable to the detection of other phosphotyrosine peptides in biological samples with minor modifications.

## Supplementary information

**Additional file 1: Table S1.** The demographic and clinical characteristics of PD patients and control individuals.

**Additional file 2: Figure S1.** Calibration curve of the heavy synthetic pY39 αβ-syn peptide in the presence of 10 ng or 2 µg of CSF peptides.

**Additional file 3: Table S2.** Quantification results for Y39 αβ-syn and pY39 αβ-syn peptides in the CSF of PD patients and control individuals.

**Additional file 4.** Skyline files of the quantification results for pY39 αβ-syn peptide in the CSF of PD patients and control individuals.

**Additional file 5: Figure S2.** Experimental strategy for the quantification of the Y39 α-syn peptide in CSF.

## Data Availability

All mass spectrometry data and search results have been deposited in the ProteomeXchange Consortium via the PRIDE partner repository with the dataset identifier PXD012202 and project name ‘Development of a method for the quantification of tyrosine 39 phosphorylated α-syn in human cerebrospinal fluid’ [16]. Reviewers can access the dataset by using ‘reviewer43678@ebi.ac.uk’ as ID and ‘jnapGY1k’ as a password.

## Abbreviations

PD: Parkinson’s disease
α-syn: α-Synuclein
β-syn: β-Synuclein
pY39 α-syn: Phosphorylation of the tyrosine residue at position 39 of α-syn
pY39 αβ-syn peptide: EGVLpYVGSK sequence
Y39 αβ-syn peptide: EGVLYVGSK sequence
TiO_2_: Titanium oxide
PTMs: Post-translational modifications
PRM: Parallel reaction monitoring
NPH: Normal pressure hydrocephalus
