# Supplementary figures and images for: Development of a novel method for the quantification of tyrosine 39 phosphorylated α- and β-synuclein in human cerebrospinal fluid
Source: Clin Proteomics. 2020 May 4;17:13. doi: 10.1186/s12014-020-09277-8 (PMC7197159; doi:10.1186/s12014-020-09277-8)

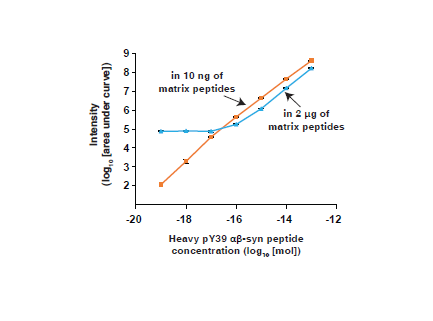

Supplement: Supplementary file 2 — Additional file 2: Figure S1. Calibration curve of the heavy synthetic pY39 αβ-syn peptide in the presence of 10 ng or 2 µg of CSF peptides. [file 12014_2020_9277_MOESM2_ESM.png]

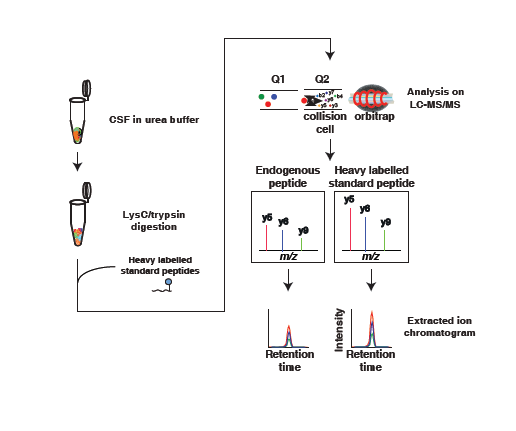

Supplement: Supplementary file 5 — Additional file 5: Figure S2. Experimental strategy for the quantification of the Y39 α-syn peptide in CSF. [file 12014_2020_9277_MOESM5_ESM.png]
